# Secondary Sulfate Effects: Schwartz Responds

**DOI:** 10.1289/ehp.10293R

**Published:** 2007-11

**Authors:** Joel Schwartz

**Affiliations:** Exposure, Epidemiology, and Risk Program, Harvard School of Public Health, Boston, Massachusetts, E-mail: jschwrtz@hsph.harvard.edu

I am not convinced by the argument of Grahame and Hidey in their letter. In fact, I find it unsupportable by modern atmospheric science and statistics. First, they argue that much of the sulfates in Boston, Massachusetts, derive locally, rather than from long-range transport, and that diesel exhaust is a probable major source. Sulfates, a major source of fine particles in Boston and other East Coast communities, derive primarily from coal-burning power plants, and this clearly may be uncomfortable for the electric utility industry and the Department of Energy; however, it is equally clear that there is little doubt about it among atmospheric scientists. For example, Salmon et al. (1997) reported that sulfate concentrations at a monitoring station in a limited-access conservation area in a rural part of western Massachusetts were almost identical to the sulfate concentrations in Boston, whereas the elemental carbon concentrations, which are markers of traffic, particularly the diesel exhaust Grahame and Hidy postulate as the source of sulfates in Boston, were quite different. The sulfate levels in Boston cannot simultaneously be driven by local traffic and still be identical to those in a distant rural area.

This similarity in sulfates persists to later periods. The U.S. Environmental Protection Agency’s Technology Transfer Network (U.S. EPA 2007) provides data on sulfate levels measured on the same days in Springfield, Massachusetts, at the western end of the state, and Boston, 95 miles to the east (Table 1). Apparently, truck traffic is almost identical in these cities.

Hourly sulfate monitors are operated in St. Louis, Missouri, Boston, and elsewhere. Hourly concentrations peak in the afternoon after the boundary layer has broken up, and transported sulfates can mix down to ground level. In contrast, concentrations of BC, a marker of diesel exhaust, peak near 0600 hours.

Regarding Grahame and Hidy’s second point, in our study (Maynard et al. 2007) we reported that the sulfate effect was no longer significant when we controlled for BC. However, because there were far fewer days with sulfate and because the coefficient was similar to the coefficient described by Laden et al. (2000), we stated that our finding strengthened the evidence for an association with mortality. We see nothing in Grahame and Hidy’s letter to lead us to change that view. Yes, the association is not statistically significant. But judgments about associations are made by combining evidence across studies, not on single studies. And in a meta-analysis, adding a study with a positive coefficient of similar size to previous studies increases the significance of the association when the cities are all combined. Thus our statement that this result adds to the support for a sulfate association remains the most reasonable interpretation. Finally, Grahame and Hidy make much of the change in effect size for sulfate when controlling for BC. Although this does indicate that there is some correlation of the measures, this correlation is to be expected, because common weather patterns drive some of the day-to-day variation in most pollutants. It does not imply that sulfate in Boston comes to a substantial extent from diesel engines.

## Figures and Tables

**Table 1:** Sulfate concentrations by year in Springfield and Boston, Massachusetts.

Year/city	No. of days	Mean (μg/m^3^)
2001		
Boston	117	3.08
Springfield	117	3.04
2002		
Boston	122	2.98
Springfield	122	2.51
2003		
Boston	124	3.03
Springfield	124	2.00
2004		
Boston	125	3.08
Springfield	125	3.01
2005		
Boston	106	3.19
Springfield	106	3.17
